# Evaluation of coronary microvascular dysfunction using magnetocardiography: A new application to an old technology

**DOI:** 10.1016/j.ahjo.2024.100424

**Published:** 2024-07-10

**Authors:** Namrita Ashokprabhu, Khaled Ziada, Edouard Daher, Leslie Cho, Christian W. Schmidt, Yulith Roca, Cassady Palmer, Sukhleen Kaur, Timothy D. Henry, Carl J. Pepine, Odayme Quesada

**Affiliations:** a Women's Heart Center, The Christ Hospital Heart and Vascular Institute, Cincinnati, OH, USA; b The Carl and Edyth Lindner Center for Research and Education at The Christ Hospital, Cincinnati, OH, USA; c Cleveland Clinic, Cleveland, OH, USA; d Ascension St. John, Detroit, MI, USA; e University of Florida, Gainesville, FL, USA; f Department of Internal Medicine, University of Cincinnati, Cincinnati, OH, USA

**Keywords:** Magnetocardiography, Coronary flow reserve, Ischemia, Coronary microvascular dysfunction, Angina and non-obstructive coronary artery disease, ANOCA, CFR, MCG, CMD

## Abstract

**Background:**

In patients with angina and non-obstructive coronary artery disease (ANOCA), diagnosis of coronary microvascular dysfunction (CMD) remains an unmet need. Magnetocardiography (MCG), is a rest-based, non-invasive scan that can detect weak electrophysiological changes that occur at the early phase of ischemia.

**Objective:**

This study assessed the ability of MCG to detect CMD in ANOCA patients as compared to reference standard, invasive coronary flow reserve (CFR).

**Methods:**

Patients with ANOCA and invasive coronary physiologic assessment using intracoronary flow measurements with Doppler and thermodilution methods were enrolled. CMD was defined dichotomously as an invasive CFR < 2.0 by Doppler or thermodilution assessment. Noninvasive 36-channel 90-s MCG scan was performed and quantitative assessment of four distinct MCG features was completed. We evaluated the diagnostic performance of 2 or more abnormal MCG features to detect CMD in the overall cohort and performed a subgroup analysis in the subset of patients with Doppler CFR assessment.

**Results:**

Among 79 ANOCA patients, 25 were CMD positive and 54 patients were CMD negative by CFR. Using invasive CFR as reference, MCG had an ROC AUC of 0.66 with a sensitivity of 68 % and specificity of 65 % for the detection of CMD. In the subgroup with Doppler CFR assessment, MCG had an ROC AUC of 0.76 with a sensitivity of 75 % and specificity of 77 %.

**Conclusions:**

In ANOCA patients, MCG demonstrates the ability to detect CMD using a 90-second non-invasive scan without the need for an intravenous stressor or ionizing radiation. Further investigations are needed to validate an MCG-based diagnostic pathway for CMD.

## Highlights

• Use of magnetocardiography (MCG) in patients with angina and non-obstructive coronary artery disease (ANOCA).
• MCG demonstrates the ability to detect coronary microvascular dysfunction (CMD) using a 90-second non-invasive scan.

## 1 Introduction

Up to 70 % of patients undergoing invasive coronary angiography have no evidence of obstructive coronary artery disease [1,2]. The majority of patients with persistent angina despite non-obstructive coronary arteries (ANOCA) have coronary microvascular dysfunction (CMD) [2]. CMD is associated with ischemia, myocardial scar and poor long-term prognosis including increased risk of major adverse cardiovascular events (MACE) and mortality [[3], [4], [5], [6], [7], [8], [9]].

The recent 2021 American Heart Association/American College of Cardiology (AHA/ACC) chest pain guidelines recommend the evaluation of CMD in patients with ANOCA [10]. However, conventional stress tests detect ischemia due to flow-limiting obstructive epicardial coronary artery disease (CAD) and have poor sensitivity and specificity for the detection of ischemia due to CMD in the absence of obstructive CAD [11]. The diagnosis of CMD is based on abnormal coronary flow reserve (CFR) acquired during rest and vasodilator-mediated hyperemia [12]. Current non-invasive modalities available to detect CMD include positron emission tomography (PET) which is the reference standard and other techniques including pharmacologic stress echocardiography and cardiac magnetic resonance imaging [13]. Invasively CFR is measured using intracoronary flow measurements with Doppler and thermodilution techniques [14,15]. These tests are limited by availability, cost, and need for pharmacologic stressor, in addition to the ionizing radiation exposure associated with PET and invasive coronary angiography [12].

Magnetocardiography (MCG) is a noninvasive imaging tool that measures the cardiac magnetic field generated by intracardiac currents from the heart's electrical activity; the very same activity which generates surface electric field potentials measured by standard 12‑lead electrocardiogram (ECG) [[16], [17], [18]]. Unlike ECG, MCG signals are undistorted by conductive tissue noise and are highly sensitive to tangential and vortex currents which are the currents induced by ischemic extracellular injury [19,20]. MCG can detect weak electrophysiological changes such as deviations in depolarization and repolarization during the ST segment interval that occur in the early phase of ischemia not detected by ECG [17,21]. Several clinical studies have demonstrated that MCG has superior sensitivity compared to ECG and other noninvasive modalities in detecting ischemic myocardium in stable CAD and acute coronary syndrome [[22], [23], [24]].

We hypothesize that MCG can detect myocardial ischemia due to CMD in ANOCA patients often missed by conventional stress tests. To test our hypothesis, we investigated the ability and accuracy of MCG to detect CMD as compared to reference-standard invasively measured CFR in ANOCA patients.

## 2 Methods

### 2.1 Study population

This trial was a multi-center prospective observational study of ANOCA patients to investigate the ability and accuracy of MCG to detect CMD as compared to reference-standard invasive CFR (NCT05150054). Between November 2021 and June 2023, eligible ANOCA patients who underwent a clinically indicated invasive coronary functional angiography (CFA) for diagnosis of CMD were recruited. Enrolled patients had a research MCG scan performed at three study sites The Christ Hospital- Cincinnati, Ohio; Cleveland Clinic- Cleveland, Ohio; and Ascension St. John Hospital- Detroit, Michigan. The protocol was approved by each site's Institutional Review Board and all patients provided written informed consent.

Patients with ANOCA who had undergone clinically indicated CFA and were diagnosed for CMD were considered for inclusion. ANOCA was defined as <50 % diameter stenosis or fractional flow reserve >0.80 in any major epicardial artery. Exclusion criteria included patients unable to complete MCG scan (active implants, non-ambulatory, or inability to lie supine/fit in the device), acute coronary syndrome within the previous 30 days, cardiomyopathy, left ventricular ejection fraction <40 %, severe valvular disease, stroke within the previous 180 days, or end stage renal disease. Data collection including demographics, clinical history, prior cardiac testing results, and current cardiac medications were extracted from the patient's medical records.

**Fig. 1:**
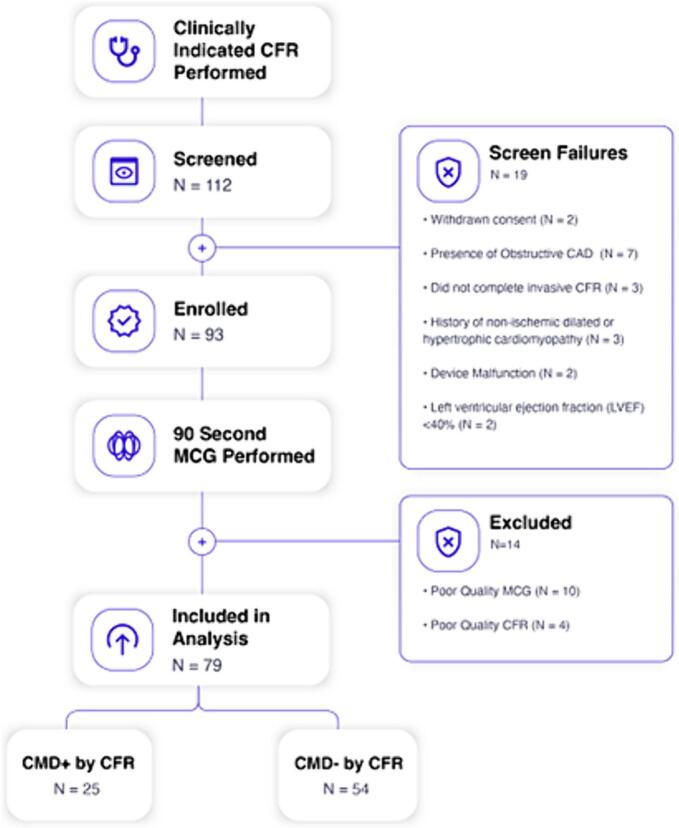
Study flow diagram. 112 patients across three sites were screened after completion of clinically indicated CFA and 19 were screen failures. Among the 93 patients enrolled in the study, 14 were excluded resulting in 79 patients in the final analysis. CAD, coronary artery disease; CFR, coronary flow reserve; CMD, coronary microvascular dysfunction; MCG, magnetocardiography.

A total of 112 patients across three sites were screened after completion of clinically indicated CFA and 93 were enrolled. Screen failures (*N* = 19) included withdrawal of consent (*N* = 2), presence of obstructive coronary artery disease (*N* = 7), missing CFR data (*N* = 3), history of non-ischemic cardiomyopathy or hypertrophic cardiomyopathy (N = 3), MCG device malfunction (N = 2), left ventricular ejection fraction <40 % (N = 2). Among the 93 patients enrolled in the study, 14 were excluded due to poor quality of MCG data (*N* = 10), or poor-quality CFR data (*N* = 4) (Fig. 1).

### 2.2 Coronary functional angiography (CFA)

All patients underwent clinically indicated outpatient diagnostic CFA via percutaneous femoral approach using standard clinical protocol. Diagnostic angiography was performed first to confirm the absence of obstructive coronary artery disease. Intravenous heparin was administered to achieve therapeutic anticoagulation. CFR was measured using the coronary Doppler technique (ComboWire XT™ or Flowire, Philips Volcano Corporation, San Diego, CA, USA) or coronary thermodilution method using pressure-temperature sensor guidewire (PressureWire X™, Abbott Vascular, Santa Clara, CA, USA) [25,26]. Steady-state vasodilator-mediated hyperemia was achieved with adenosine [27]. For the Doppler technique the Doppler-tipped guidewire was positioned in the proximal LAD and hyperemia achieved using 72 μg intracoronary adenosine bolus injections. CFR is calculated as hyperemic flow velocity divided by resting flow velocity. For the thermodilution method, the pressure-temperature sensor guidewire was positioned in the mid-distal LAD and continuous intravenous adenosine infusion of 140 μg/kg/min was used to achieve hyperemia [14]. CFR is calculated as the resting mean transit time divided by the hyperemic mean transit time. CMD positive was defined as CFR <2.0 by Doppler or thermodilution assessment and reference group (CMD negative, CFR ≥2) [28].

### 2.3 Magnetocardiography (MCG)

After invasive CFA, enrolled patients had an MCG scan performed using a 36-channel magnetometer system (CardioFlux Magnetocardiograph; Genetesis Inc., Ohio, USA) as an outpatient study procedure. Following standard operating protocol, study patient was placed in the supine position on a bed and the MCG operator positioned the sensor array directly above the patient's chest (approximately 1-cm above the anterior chest wall) and then inserted the subject's bed into a small open-ended magnetic shield. 90-second MCG measurements were performed at room-temperature using contemporary optically pumped magnetometers and at rest without the use of pharmaceuticals (i.e. vasodilators, radiotracers, inotropic agents) or exercise. Cardiac medications were not held for the test.

**Fig. 2:**
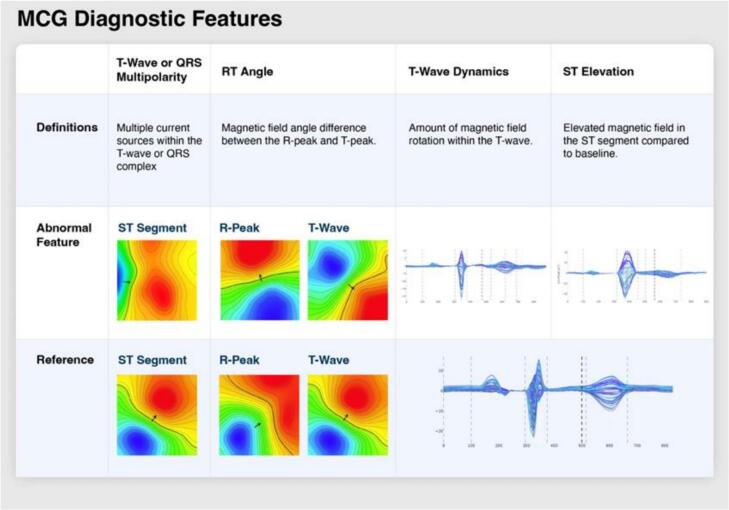
Magnetocardiography diagnostic features for coronary microvascular dysfunction. (1) ST island – monopolar field map patterns in the ST segment; (2) RT angle – a significant angular difference between the orientations of the R peak and T wave magnetic field maps; (3) ST dynamics – correlated collective variation of channels in the ST segment waveform; (4) ST elevation – excess electromagnetic energy in the ST segment.

In the past decade, advancements in sensor technology have allowed for a transition from superconducting quantum interference device (SQUID) measurements of MCG to room-temperature optically pumped magnetometer measurements [29]. These advancements, in addition to the advent of modern computing, have allowed for more clinically practical MCG measurements to be made focused on quantitative features of the magnetic field during the ST interval analogous to ECG-based repolarization analysis [30]. Quantitative assessment of four distinct MCG features, with equal weights, of the cardiac waveform and magnetic field map (MFM) – RT angle, ST island, ST dynamics and ST elevation were used to interpret MCG scans (Fig. 2). These four distinct MCG features are defined as follows: (1) ST island – monopolar field map patterns in the ST segment; (2) RT angle – a significant angular difference between the orientations of the R peak and T wave magnetic field maps; ST dynamics – correlated collective variation of channels in the ST segment waveform; (4) ST elevation – excess electromagnetic energy in the ST segment. Heterogeneous repolarization due to ischemia is reflected in the ECG through changes in the ST-segment, such as ST segment elevation or depression, and T-wave changes such as inverted T wave, biphasic T wave. These ischemia-related changes increase the signal entropy of beat-to-beat ST-T segments, leading to morphological variabilities captured in the four distinct MCG features selected. ECG and MCG signals share ischemic temporal changes, but MCG uniquely shows the spatial changes – as demonstrated by these MCG features – in the form of the cardiac waveform and MFM [[16], [17], [18]]. The MCG signal is typically in the picoTesla (pT) range, millions of times smaller than the ambient magnetic field of the Earth. The frequencies of interest for the MCG range from 0.5 Hz to 50 Hz, with some explorations of low-frequency signal occurring at the direct current level [17].

A separate registry of healthy, asymptomatic patients without history of cardiac disease (*N* = 26) was used to calibrate the cutoff values for each of the MCG features. The cutoff for each MCG feature was set at the 85th percentile within this population. Each of the MCG features was associated with a quantitative score that is automatically extracted from the MCG scan data and was flagged as abnormal if exceeded the 85th percentile cutoff value and considered normal otherwise. Each patient was assigned an MCG score between zero and four based on the number of abnormal MCG features. The treating physicians and research participants were blinded to the MCG scan results.

### 2.4 Statistical analysis

Values are expressed as mean (standard deviation), median (Quartile 1 [Q1], Quartile 3 [Q3]) and frequency (percentages) as indicated. Statistical differences between the CMD positive and CMD negative groups (as defined by invasive CFR) were calculated using the Wilcoxon rank-sum test for continuous variables and the Chi-square and Fisher's exact test for categorical variables, as appropriate.

The diagnostic performance of MCG for detecting CMD (as defined by invasive CFR) based on the number of abnormal MCG features was assessed by evaluating the sensitivity, specificity, positive predictive value (PPV), negative predictive value (NPV) and the area under the receiver-operating characteristic curve (ROC AUC) with 95 % confidence intervals. To provide a balanced test, CMD positive by MCG was defined as two or more abnormal MCG features out of the four distinct features.

A subgroup analysis was performed in patients with Doppler CFR assessment (*N* = 54) using the same cutoff of CFR <2.0 to define CMD by Doppler. Sensitivity, specificity, PPV, NPV, and ROC AUC analyses were performed for this subgroup. For all analyses, a *p*-value of <0.05 was considered statistically significant. All analyses were performed using Stata version 17.0 (College Station, TX.)

## 3 Results

### 3.1 Study population

Among the 79 patients that were included in final analysis, 32 % (*N* = 25) were CMD positive by CFR and 68 % (*N* = 54) were CMD negative by CFR. The median (Q1, Q3) age of patients was 59 (50, 65) years and the majority of the cohort was female (85 %), White race (90 %) and non-Hispanic ethnicity (97 %) (Table 1). Cardiovascular risk factors were prominent in the overall cohort including 87 % with hyperlipidemia, 71 % with hypertension, 14% with diabetes, 44 % with family history of cardiovascular disease, and 30 % current or former smoker. CMD positive and CMD negative patients were similar in age and in their cardiovascular risk profile.

**Table 1:** Demographics and clinical characteristics in patients with and without coronary microvascular dysfunction.

Variables	Overall cohort (n = 79)	CMD negative (n = 54)	CMD positive (n = 25)	*P* value
Demographics				
Age, yr, median (IQR)	59 (50, 65)	57 (46, 64)	61 (58, 65)	0.023
Female sex, n (%)	67 (85)	44 (81)	23 (92)	0.320
Race				
Black, n (%)	8 (10)	5 (9)	3 (12)	0.703
White, n (%)	71 (90)	49 (91)	22 (88)	0.703
Ethnicity				
Hispanic, n (%)	2 [40]	1 (2)	1 (4)	0.541
Non-Hispanic, n (%)	76 (97)	52 (98)	24 (96)	0.541

Cardiovascular risk factors				
BMI, kg/m^2^, median (IQR)	30.3 (24.8, 36.9)	29.3 (24.9, 36.9)	30.9 (24.3, 36.4)	0.985
Diabetes, n (%)	11 (14)	7 (13)	4 (16)	0.735
Hypertension, n (%)	56 (71)	35 (65)	21 (84)	0.111
Hyperlipidemia, n (%)	69 (87)	47 (87)	22 (88)	1.000
Current or former smoker, n (%)	24 (30)	19 (35)	5 (20)	0.172
Family history of CVD, n (%)	35 (44)	21 (39)	14 (56)	0.154

Medications, n (%)				
Beta blockers	45 (57)	26 (48)	19 (76)	0.020
Calcium channel blockers	50 (63)	32 (59)	18 (72)	0.275
Nitrates	61 (77)	41 (76)	20 (80)	0.688
Aspirin	56 (71)	36 (67)	20 (80)	0.225
Statins	62 (78)	39 (72)	23 (92)	0.075
ACE-I/ARB	36 (46)	20 (37)	16 (64)	0.025

Prior cardiac testing				
Any prior positive stress test, n (%)	27 (34)	19 (35)	8 (32)	0.781
Number of stress tests, mean (SD)	1.8 (1.2)	1.8 (1.1)	2.0 (1.3)	0.579
Number of invasive angiographies, mean (SD)	1.0 (1.5)	0.9 (0.9)	1.4 (2.3)	0.114

Invasive CFA				
Thermodilution method, n (%)	25 (32)	20 (37)	5 (20)	0.130
Doppler method, n (%)	54 (68)	34 (63)	20 (80)	0.130
CFR, median (IQR)	2.6 (1.8, 3.2)	2.9 (2.5, 3.6)	1.6 (1.4, 1.8)	<0.001
Time between CFR and MCG, days, median (IQR)	26 (13, 41)	27 (12, 47)	24 (15, 32)	0.377

ACE-I/ARB, Angiotensin-converting enzyme inhibitors/angiotensin receptor blockers; BMI, body mass index; CFA, coronary functional angiography; CFR, coronary flow reserve; CMD, coronary microvascular dysfunction, MCG, magnetocardiography. CMD positive defined as invasive CFR < 2.0 by Doppler or Thermodilution methods.

The CMD positive cohort had significantly higher rates of medications prescribed pre-CFA including beta blockers, and angiotensin converting enzyme inhibitors (ACEI) or angiotensin reception blocker (ARB) (Table 1). The rates of aspirin, statins, calcium channel blockers, and nitrates were similar between the groups. On average both groups had similar number of stress tests performed prior to CFA, similar rates of prior positive stress test for ischemia, and invasive coronary angiography completed prior to CFA. The majority of CFA was performed using Doppler method (68 %). The median (Q1, Q3) CFR was significantly lower in the CMD positive group at 1.6 (1.4, 1.8) compared to 2.9 (2.5, 3.6) in the CMD negative group (*p* < 0.001). The median (Q1, Q3) time difference between the invasive CFA and MCG scan was similar between the groups and on average 26 (13, 41) days.

### 3.2 MCG performance

Using invasive CFR as reference, the sensitivity, specificity, PPV, and NPV based on the number of abnormal MCG features is shown in Table 2. A single abnormal MCG feature is associated with a high sensitivity (92 %) cutoff and three or four abnormal features with a high specificity (83 % and 98 % respectively). Using invasive CFR as reference, 2 or more abnormal MCG features had an AUC ROC of 0.66 (95 % CI 0.55, 0.78) with a sensitivity of 68 % (95 % CI 47, 85), specificity of 65 % (95 % CI 51, 77), NPV of 81 % (95 % CI 67, 92) and PPV of 47 % (95 % CI 30, 65) for the detection of CMD in the overall cohort (Table 3). In subgroup analysis, in the subset of patients with Doppler assesment (54 patients including 20 CMD positive), MCG had an AUC ROC of 0.76 (95 % CI 0.64, 0.88) with a sensitivity of 75 % (95 % CI 51, 91), specificity of 77 % (95 % CI 59, 89), NPV of 84 % (95 % CI 66, 95) and PPV of 65 % (95 % CI 43, 84).

**Table 2:** Magnetocardiography performance for evaluation of coronary microvascular dysfunction by number of abnormal MCG features.

Number of abnormal MCG features	N	Sensitivity (95 % CI)	Specificity (95 % CI)	NPV (95 % CI)	PPV (95 % CI)
1	63	92 (74–99)	26 (15–40)	88 (62–98)	37 (25–50)
2	36	68 (47–85)	65 (51–77)	81 (67–92)	47 (30–65)
3	13	16 (5–36)	83 (71–92)	68 (56–79)	31 (9–61)
4	2	4 (0−20)	98 (90–100)	69 (57–79)	50 (1–99)

**Table 3:** Performance of magnetocardiography (2 or more abnormal features) for evaluation of coronary microvascular dysfunction in patients with angina with no obstructive coronary artery (ANOCA).

Diagnostic test	N	Sensitivity (95 % CI)	Specificity (95 % CI)	NPV (95 % CI)	PPV (95 % CI)	AUC (95 % CI)
Overall cohort	79	68 % (47–85)	65 % (51–77)	81 % (67–92)	47 % (30–65)	0.66 (0.55–0.78)
Doppler method only	54	75 % (51–91)	77 % (59–89)	84 % (66–95)	65 % (43–84)	0.76 (0.64–0.88)

## 4 Discussion

In this first-of-its-kind study, we demonstrate MCG's ability to detect CMD in ANOCA patients using a 90-second non-invasive scan without intravenous stressor or ionizing radiation. The ability of MCG to detect CMD was moderate in the overall population, but improved markedly for patients with invasive Doppler CFR assessment.

MCG has been extensively studied for the diagnosis of myocardial ischemia and shown to be a promising alternative to stress testing in patients with obstructive coronary artery disease; and more recently in patients with stable CAD using a rest scan [21,31]. In this study, we extend these findings to the ANOCA population. We demonstrate that MCG is highly sensitive (92 %) to detect CMD in an ANOCA population when one abnormal MCG feature is detected and highly specific (83 %–98 %) when three and four abnormal MCG features are detected. The high sensitivity and NPV of MCG to detect CMD in an ANOCA population mirrors that of stress testing for diagnosis of obstructive flow limiting CAD [32]. Two abnormal features resulted in a balanced level of sensitivity and specificity with an ROC AUC of 0.66. MCG had significantly better performance in patients with CMD diagnosed by Doppler-derived CFRs with an ROC AUC of 0.76. The better performance seen in the subgroup with Doppler-measured CFR is likely because thermodilution method overestimates CFR [33]. Our results are consistent with non-invasive gold-standard PET myocardial perfusion which was found to correlate significantly better with Doppler measured CFR (*r* = 0.82, *p* < 0.001) than thermodilution (*r* = 0.55, p < 0.001) [40]. Our findings are also in line with the performance of vasodilatory stress cardiac magnetic resonance imaging visual assessment (ROC AUC 0.6, 95 % CI 46 % to 69 %) and quantitative myocardial perfusion reserve index (ROC AUC 0.88, 95 % CI 95 % to 96 %; sensitivity 70 % and specificity 90 %) as compared to CFR [34].

MCG is able to detect ischemia-induced extracellular injury currents which are predominantly tangentially oriented and not detected by ECG [35]. The Women's Ischemia Syndrome Evaluation Coronary Vascular Dysfunction Study (WISE-CVD) study showed in a cohort of ANOCA patients that myocardial ischemia was detectable in all patients using an ultra-high sensitivity cardiac troponin assay collected at a random time point suggesting a chronic low-grade myocardial ischemia irrespective of symptoms in these patients[4,6]. The WISE-CVD study also showed evidence of myocardial scar on cardiac magnetic resonance imaging in a significant proportion of ANOCA patients consistent with recurrent ischemic cardiomyocyte injury [9]. Further, Ahmad et al. demonstrated in a cohort of 1552 ANOCA patients who underwent invasive CFA, those with CMD had signature ECG changes at rest (T wave repolarization parameters) suggestive of chronic myocardial ischemia at rest [36]. We hypothesize that a rest MCG scan is able to detect the complex electrophysiologic alterations associated with chronic low-grade myocardial ischemia in patients with CMD that remain to be fully understood. Our hypothesis is supported by prior work demonstrating detectable altered MCG signals at rest in a subset of patients with low degree of coronary artery stenosis [21].

**Fig. 3:**
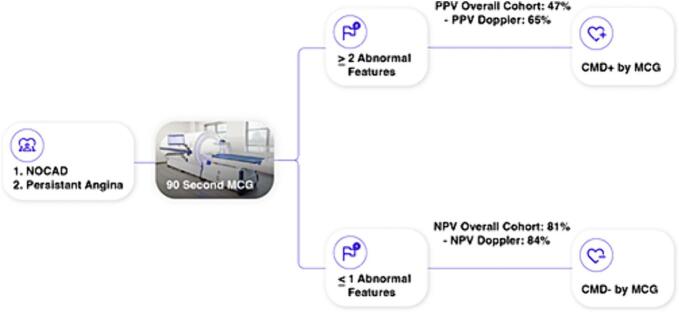
Central illustration: magnetocardiography diagnostic pathway for coronary microvascular dysfunction. An MCG diagnostic pathway for CMD includes the use of 2 or more abnormal MCG features to evaluate patients with ANOCA and suspected CMD. NOCAD, non-obstructive coronary artery disease; MCG, magnetocardiography; CMD, coronary microvascular dysfunction; PPV, positive predictive value; NPV, negative predictive value.

The Stratified Medical Therapy Using Invasive Coronary Function Testing in Angina trial (CorMicA) recently demonstrated that management based on microvascular dysfunction endotypes resulted in significant improvement in quality of life among ANOCA patients [37]. However, in the United States (U.S.) invasive physiological testing with CFA in the ANOCA population is rarely performed at the time of diagnostic angiogram and CFA is limited to specialized centers resulting in underdiagnosis and undertreatment of CMD, which is associated with significant economic burden [38]. Coronary computed tomography angiography (CCTA) is emerging as a first-line, effective and rapid diagnostic modality to evaluate for obstructive coronary artery disease [39]. However, CCTA is limited to anatomical and functional assessment (fractional flow reserve) of the epicardial arteries. Therefore, accessible noninvasive testing methods that are easy to perform are essential to fill the unmet clinical need for the initial assessment of patients with ANOCA. We propose a diagnostic pathway that includes the use of 2 or more abnormal MCG features to evaluate for CMD in patients with ANOCA where obstructive coronary disease has been ruled out with prior diagnostic angiogram or CCTA (Fig. 3). However, it is important to note that in patients with ANOCA and vasospastic angina, invasive coronary physiology assessment is warranted for the evaluation of microvascular spasm and coronary vasospasm [15].

There are strengths and limitations of this study. MCG was previously hindered by shielding requirements, such as magnetically shielded rooms and liquid helium for cooling, sensor limitations and recurring costs. However, advances in technology have led to ability to operate at room temperature in a semi-shielded environment greatly expanding accessibility. MCG is precise and has superior reproducibility to any electrode system given the fixed sensor positioning relative to the patient [20]. There are no recurring costs or specialized training required to operate the device or need for physician supervision since the MCG scan can be done at rest. Despite these benefits, the study has limitations that should be noted. The sample size was relatively small, but comparably sized to other noninvasive imaging studies in the ANOCA patient population with sample sizes between 21 and 75 patients [11,34]. There was a mean time difference of 26 days between the invasive CFA and MCG scan; however, this amount of time is not expected to result in meaningful improvement in CFR. Notably, 11 % of participants were excluded due to poor MCG quality therefore further technological advancements are needed for clinical adaptability. Additionally, the correlation between index of microcirculatory resistance (IMR) and MCG was not assessed as data was only available for a small subset of patients (*n* = 25). Further, this study was designed to be hypothesis-generating, and accordingly, was not powered to demonstrate efficacy; therefore, an adequately powered study, with further exploration of MCG variables, for the diagnosis of CMD is currently in development.

## 5 Conclusions

This study provides the first reported data on the use of MCG for the detection of CMD in ANOCA patients. As compared to the reference-standard invasive CFR, MCG can detect CMD with comparable efficacy to currently available non-invasive diagnostic modalities in a rapid, safe, noninvasive assessment without ionizing radiation. Adequately powered prospective studies are needed to determine MCG's role in the diagnosis of CMD in ANOCA patients.

## Clinical Implication

• Diagnosis of CMD remains an unmet need in ANOCA patients.
• MCG detected CMD using a non-invasive scan without the need for an intravenous stressor or ionizing radiation.
• Further investigation as needed to validate an MCG based diagnostic pathway for CMD in ANOCA patients.

## CRediT authorship contribution statement

**Namrita Ashokprabhu:** Methodology, Formal analysis, Conceptualization. **Khaled Ziada:** Methodology, Investigation, Data curation. **Edouard Daher:** Methodology, Investigation, Formal analysis, Conceptualization. **Leslie Cho:** Visualization, Validation, Formal analysis, Data curation. **Christian W. Schmidt:** Data curation, Conceptualization. **Yulith Roca:** Writing – review & editing, Writing – original draft, Data curation, Conceptualization. **Cassady Palmer:** Software, Resources, Methodology, Data curation. **Sukhleen Kaur:** Methodology, Investigation. **Timothy D. Henry:** Writing – review & editing, Writing – original draft, Formal analysis, Conceptualization. **Carl J. Pepine:** Writing – review & editing, Writing – original draft. **Odayme Quesada:** Writing – review & editing, Writing – original draft, Supervision, Investigation, Funding acquisition.

## Declaration of competing interest

Odayme Quesada reports financial support was provided by National Heart Lung and Blood Institute Health Information Center. Khaled Ziada reports a relationship with Abbott Vascular Inc. that includes: consulting or advisory. Carl Pepine reports a relationship with Amgen Inc. that includes: funding grants. Carl Pepine reports a relationship with BIOCARDIA, INC. that includes: funding grants. Dr. Ziada receives honoraria from Abbott Vascular, Inc. and financial support from Medtronic, Inc. for meetings and/or travel. Dr. Pepine receives grant funding from Amgen, BioCardia, Inc.; Brigham & Women's Hospital; CLS Behring; DoD-CDMRP/WARRIOR Trial; DoD PRMRP/QUIET WARRIOR Study; Gatorade Trust; GE Healthcare; McJunkin Family Foundation Trust; National Institutes of Health/National Heart, Lung and Blood Institute; National Institutes of Health/NIA; National Institutes of Health/NIHAAA; and Sanofi-Aventis. Dr. Pepine is a consultant for BioCardia, Inc.; Elsevier/American Heart Association Journal Plus; Janssen Pharmaceuticals; Sanofi-Aventis; Healio/Wyanoke/Cardiology Today; and XyloCor Therapeutics, Inc. Dr. Pepine receives financial support from the University of Florida for meetings and/or travel. Dr. Pepine serves on a Data Safety Monitoring Board (DSMB) or Advisory Board for Verily Life Sciences, LLC. Project Baseline OSMB via DCRI. Dr. Quesada received financial support from Genetesis, Inc. for meetings and/or travel. If there are other authors, they declare that they have no known competing financial interests or personal relationships that could have appeared to influence the work reported in this paper.

## Data Availability

The data underlying this article will be shared on reasonable request to the corresponding author.
